# Analytic Study of Cantharis

**Published:** 1889-03

**Authors:** Edward Fornias

**Affiliations:** 711 Pine Street, Philadelphia


### ANALYTIC STUDY OF CANTHARIS.

| | | |
|---|---|---|
| CANTHARIS,<br><br>< Drinking cold water and coffee.<br><br>> Rubbing, warmth, and lying down.<br><br>/\ Coffea. | URINARY TRACT. | 1. Irritation, inflammation, *ardor urinæ, tenesmus versicæ*, cystospasmus, *priapismus, chordee, dysuria, stranguria* (Caps.), *hæmaturia*, strong sexual desire (Phos.), *cutting, burning in urethra before, during, and after urination* (Can. sat.). Also stinging, dull aching, crampy, tearing, jerking, drawing, and pulsative pains; paroxysmal as a rule, either on the renal region, along the ureters, down to the bladder, urethra, and even to tip of penis; limited to the bladder, urethra, spermatic cord, and even testicles. *Scanty, bloody, albuminous urine, containing cylindrical casts*, mucus shreds, or pus; or turbid, like loam water. It passes in a thin, divided stream. (Can. sat.) Discharge of dirty, purulent, or yellow fluid from the urethra, often mixed with blood. Swelling of the glands. |
| | DIGESTIVE TRACT. | 2. Furred tongue, with red edges; swollen, *excoriated, trembling* (Lach.). Sponginess, dryness, soreness, and burning of the oral mucosa. *Thirst, with aversion to all fluids, especially water* (Ars.); even the sight of water makes the patient worse. Dysphagia, *Pharyngeal spasms, especially when attempting to swallow water.* Vomiting, with violent retching. *Sensitiveness and violent burning in stomach* (Ars., Caps.). Burning, cutting, *great distention and painful sensitiveness of the abdominal walls to touch.* Pain in the bowels and heat in the intestinal canal. *Dysenteric stools, consisting of white mucus and solid pieces, looking like false membranes* or *scrapings of intestines* (Colch., Coloc.). Green or bloody-mucous stool, < at night. preceded by colic, attended by burning at anus, and followed by *tenesmus, burning, and chilliness* (Merc. cor.). |

CANTHARIS.

RESPIRATORY TRACT.

3. *Dryness and weakness of the air-passages, with feeble, timid voice.* Soreness of the larynx. Contraction, constriction, or burning heat in the larynx and trachea (Caps.). *Hoarseness.* Roughness of throat and chest. Early morning cough, with difficult expectoration. *Short, dry, hacking cough.* Bloody expectoration after a short spell of coughing. *Hawking,* with detachment of tenacious, viscid, or bloody mucus. Difficult breathing, even dyspnœa. *Pressure, tension,* or *stitches now here, now there,* in *chest, especially in the right side.* Sticking pain and stitches in lower right chest, extending toward the middle of the sternum, sometimes on inspiration (Bry., Kali c.). Stitches on the left side, at night, during inspiration, not permitting one to lie on that side, with arrest of breathing. Heat and burning in chest, with rising of little clots of blood, or darting pains in chest. Chest very sensitive to touch. *Exudation within the pleura.*

SKIN.

4. *Tingling, heat, smarting, burning.* Reddened and raised papillæ. *Vesicles and blebs of various sizes,* filled with a yellow-white serum, rich in albumen, assuming later a purulent character. Redness and swelling of the underlying tissues. *Ulceration.* Gangrene. *Erysipelatous inflammation of vesicular type* (Rhus tox.). Principally in the nose, face, hands, arms, and chest. *In the dorsum of the nose spreading to both cheeks, but more to the right, with swelling, hardness, and subsequent desquamation.* Ulcerative pain in the affected parts when touched. Ulcers, with itching and lacerating pains, especially in the legs. Eczema. Erythema.

In order to study this drug advantageously, we must analyze the sections of the summary one by one, and commencing with the genito-urinary organs, we perceive at once how profound and marked is its action on that especial sphere. From a slight irritation it advances to violent inflammation, and even destruction of the parts involved. The accompanying symptoms are of an acute and painful character, bringing about great suffering and distress. This important group clearly reveals the applicability of the remedy to *urethritis, cystitis, nephritis, and other*

*urinary difficulties. Even retention and suppression of urine, with uræmic coma, delirium, and convulsions* are conditions often demanding this drug. But we must bear in mind that all the symptoms exhibited by this drug elsewhere are almost subordinate to the *urinary phenomena*, which are, we may say, its leading feature.

In *specific urethritis*, no matter in which stage, it is indicated when the inflammation threatens to extend, or does actually extend to the bladder. Its selection here does not depend so much on the character of the pain and discharge, as upon the *dysuric* or *stranguric symptoms. Painful erections, priapism* and *satyriasis*, as well as *chordee* and *hæmaturia* are complications which prominently point to this remedy. But the most dreadful complication is *cystitis*, either from acute or suppressed gonorrhœa, for in such cases the disease is prone to involve the prostate gland, and in severe cases extend to the kidneys. *The twisted or divided stream, the nocturnal urgings, and the difficulty of passing water, with discharge of a few drops of blood instead of urine*, indicate this remedy in *stricture of the urethra*, either the spasmodic or callous, especially after gonorrhœa. It may be even advantageously employed in *morbis Brightii*, if the symptoms agree. In *hæmaturia* the discharge is either of fluid blood, in drops, or coagulated, with violent urging and tenesmus. The scanty loss of blood is usually attended by cutting and crampy pains in the bladder, extending up to the kidneys and down the urethra. *Painful burning in the urethra after coitus* is a condition I had occasion to treat successfully with *Cantharis.* We must just here remember the *itching and burning of the pudendum and the inflammatory swelling of the cervix and os uteri*, symptoms to be considered in treating gonorrhœa of the female.

The various pains produced by *Cantharis* have been grouped together in order to study them more conveniently. The most prominent and constant are *cutting* and *burning*, which in the *urethra especially* are exhibited with characteristic persistence. They not only precede and accompany the act of micturition, but remain for some time after, decreasing and again increasing in gravity, and thus assuming a *paroxysmal character*. The *cutting* especially is sometimes so violent as to cause one to bend double. The urethra is not their only site, we find them in the renal region, running from kidneys to ureters and bladder; from the neck of the bladder extending to the fossa navicularis; along the spermatic cord to testicles, and down the penis, with drawing up of the testicles; in the ovaries, and even in the abdominal walls and viscera. But *burning and cutting* are not the only pains produced and cured by *Cantharis*. In the *kidneys* we have also *stinging, tearing, pulsative, and pressing pains*. In the *renal region, a dull aching or jerking*. Along the *ureters, tearing and contractive pains*. In the *bladder*, together with the *burning and painful urging*, there is a marked *stinging sensation*, as well as *crampy pains*, the latter extending up to kidneys and down to the urethra. In the *neck of the bladder, pressing, tearing, and stinging, and at its forepart, stitching pain*. In the *urethra, tingling, jerking, biting, and tension*. The *tingling* especially after micturition. Also *itching before and after passing water*. In the *spermatic cord, drawing and jerking*.

The knowledge of the nature and site of so many painful phenomena is obviously important.

Passing now to the second group of the summary, a glance at it will suffice to value rightly its especial adaptability to *gastritis, enteritis, peritonitis, and dysentery*, and this would still become more patent should the bladder become involved in the morbid process.

In *acute gastritis, a thickly-furred tongue, with red edges; the aversion to all kinds of food; a great thirst, yet loathing of drink; sour eructations; nausea and vomiting of water drunk, of bile, or ingesta, with violent retching; occasional dysphagia and aphonia; chills or rigors, and, above all, the sensitiveness and violent excoriating burning pain in the stomach, especially if the patient tosses about, as if in despair, on account of the pain*, are all symptoms indicative of *Cantharis*. If the inflammation of the stomach is due to the ingestion of irritant and corrosive poisons, and after administering the proper antidotes there should remain *vomiting of shreds of mucous membranes and purging of the same nature, with an intense burning pain referred to the stomach; cold skin; clammy sweat; weak and timorous speech; death-like features; internal heat and thirst, with aversion to water; variable pulse, and finally weakness, prostration, and faintness, Cantharis* becomes again the remedy.

The *discharge of membranous shreds or casts from the bowels*, with or without blood, being the characteristic feature of *croupous enteritis*, we cannot well overlook *Cantharis* in this affection, especially since we know that in such cases the passage of necrosed mucous membrane is usually attended by cutting pains and tenesmus, and the abdomen is very tender to touch.

No less useful is the remedy in *peritonitis*, a disease which not only exhibits *marked abdominal tenderness, tympanitis, and violent cutting pains*, but which by extension may implicate the bladder, and produce *strangury and vesical irritation*. This drug would still become more prominently indicated should the patient fall at once into collapse, a condition usually present in *peritonitis from perforation.*

But it is in *acute dysentery* or *ulcerative colitis* where *Cantharis* has been employed with great success. In the *sporadic variety the bloody stools, the over-production of mucus, the cutting pains in the abdomen, the burning in the rectum and the almost constant tenesmus* have been the leading symptoms for its employment; while in the *croupous* or *epidemic form* more than the pains and tenesmus, it is the presence in the stools *of blood* and *epithelial debris mixed with mucus,* that first led us to this drug in this painful affection. We must also remember that *nausea and vomiting, strangury, anxious countenance, expressing extreme suffering; sunken eyes, surrounded by livid circles; cold skin, feeble pulse, faintness, chilliness, prostration, and collapse,* all symptoms of *Catharis,* are usual concomitants of this disease.

Entering now upon the consideration of the *third group,* we can but admit that in this country we have not brought to advantage all the varied symptoms produced by *Cantharis* in the *respiratory tract.* French homœopathists, and among them Dr. Chargê, have not only employed this drug successfully in *pleuritis,* but in *pulmonary congestion* and even *aphonia.* The said Doctor, in his work entitled "*Traitement Homœopathique des Maladies des Organes de la Respiration,*" recommends this remedy in *aphonia* following quinsy with hoarseness, when we can admit, as near causes, thickening of the mucosa of the larynx and atony of the nerves, or when it is sympathetic to an affection of the *urinary tract.* Also *in pulmonary congestion,* when there is *burning in chest, difficult and accelerated breathing; oppression; burning pain in the chest, with a little blood in the sputa; stitches in the chest from one side to the other, which manifest themselves and usually become worse on inspiration; precordial anxiety, palpitation and stitches in the heart; cough provoked by a tickling in the throat, with oppression and accelerated respiration.*

According to this authority, the most favorable time for its employment *in pleuritis* is when the fever subsides, and the exudation persists or increases. "It is counter-indicated when the *pulse is hard, strong, and about* 100, *and when the pain in the side is very acute. The fever of Cantharis is attended principally by coldness and chilliness. The pulse is small, contracted, hard, and somewhat frequent, but there is no external heat. There is paleness about the nose and mouth, and blue rings about the eyes. The dullness is complete; absence of vesicular murmur; tubular souffle, stronger on expiration than on inspiration, in the*

*apex of the lung, front and back, intense dyspnœa; palpitation; wet skin or profuse sweat; restless nights.*"

"*The cough is short, dry, and frequent. Painful stitches arresting the respiration, principally in the sides, right and left, attended by more or less dyspnœa.*"

"If, with all these signs of an exudation, which by itself is sufficient to determine the choice of *Cantharis*, the patient should exhibit as concomitants any of the following symptoms: *Tongue peeled off, very painful and covered with small flat ulcers; lower abdomen sensitive to touch; urine scanty, with painful and frequent emissions; great debility, and tendency to syncope*, we have then the complement for the perfect indication of this drug, which in such cases will prove to be the remedy *par excellence.* But, of course, no matter how real the beneficial results obtained by this new therapeutic agent may be, we should not extol it in detriment of *Sulphur*, which in pleuritic exudations stands uppermost."

It is an indisputable fact that whatever success the old school has attained by the local use of the *Spanish-fly* in the treatment of *pleuritis*, it has been due to its dynamic action, for blisters, if we are to believe those who are in the habit of using them, are only beneficial in pleuritis when the fever decreases, the pain lessens or disappears, or the exudation persists or threatens to increase. To consider even the fallacious notion that free vesication and the maintenance of the discharge by irritating ointment will drain off the fluid, as it were, from the water-logged pleura, would be unpardonable.

This barbarous treatment, says even Ringer, drains important nutritive material from the system and weakens the patient when strength is most needed. We might just as well bleed to the same amount, for the serum of blisters contains almost as much albumen as the blood itself.

It is very significant that many of our opponents admit the fact that blisters will redden and inflame the pleura, and condemn the practice of blistering to the point of vesication, asserting that it depresses the bodily powers in proportion to the amount of serum withdrawn from the vessels, and still they believe that by blistering in a milder way good results can be obtained. If this is the case, it must be by virtue of the dynamic action of the remedy.

How often have some of our men overrated the value of *Bryonia* by simply ignoring or forgetting the fact that in this acute affection the severe pain is of short duration and spontaneously lessens or disappears.

The phenomena developed on the *skin* and comprised in the *last group* are principally the effects of the local action of *Cantharis.* Its irritative action here results in an *erysipelatous inflammation of the vesicular type.* As soon as a blister is applied to the surface of the body we experience *tingling, smarting, and a sensation of heat; the papillæ quickly become reddened and raised; next, in a variable time determined by the strength of the application, on these papular elevations minute vesicles form, which gradually enlarge, and by their lateral extension soon coalesce, so as to form blebs of various sizes, filled with a fluid rich in albumen, and generally contain some fibrine, later assuming a purulent character. If allowed to act for a long time, ulceration and destruction of the tissues will ensue,* or in other words, if blistering is carried far enough to produce large blebs, the serum will not become absorbed and the blebs will at last burst. Should the air then penetrate the raw surface, a violent inflammation sets in, which may end in an extensive slough. Such effects should be borne in mind when treating deep, severe burns.

I could not close my remarks on this drug, without making even allusion to its applicability in diseases having their point of departure in the cerebro-spinal system, such as *rabies, spasm, tetanus,* etc. The congestion and inflammation of the brain, attended by *delirium, stupor, and dilated pupils,* advancing to *coma,* and ending finally in *death,* clearly show its powerful action on this region; while the *prostration, powerlessness, dysphagia, dread of liquids, frightful convulsions, tetanus, collapse, and death* point to its action on the cord.

Through this same system, this drug acts also upon the *heart and circulation,* first as a *stimulant,* secondly as a *depressant,* the order of these effects depending largely upon the dose. In the first case under its stimulus *the pulse is quickened,* the *arterial tension increased, and the temperature raised;* while on the second, *a lowering of the pulse and arterial tension and a decline of the temperature mark the advancing depression.*

Its action on the *mind* seems to be in harmony with its primary and secondary general effects. *Apprehension, anxiety, restlessness, irritability, nervousness, vehemence, insolence, contradiction, frenzy, and rage,* on the one side; *dullness, languor, confusion, distraction, and despondency* on the other. Of all these varied mental conditions, the most characteristic are the *rage and frenzy;* the former *paroxysmal in nature, commencing usually with an anxious restlessness, attended by crying, barking, and beating, and renewed by the sight of water or other bright, dazzling objects, when touching the larynx, and on attempting to drink*

*water* (a complete picture of *hydrophobia*); the latter, *amorous in character, with strong and persisting erections and an irresistible sexual desire.* Next in importance comes the *confusion, distraction, irritability, and despondency. The confusion is attended by pulsation in the forehead and occurs principally in the morning. The patient is so distracted that he is unable to concentrate thought, so irritable that he is dissatisfied with every one and everything, so despondent, that she says she must* die. And finally in *insolence and contradiction* compares favorably with the Nux vom. patient. With *Bell., Hyosc.*, and *Stram., Cantharis* forms an important group to combat *a furious, almost frenzied delirium.*

The pernicious effects of *Cantharis* are readily neutralized by *Camphor*, with which it bears many points of resemblance. Its other antidotes are *Acon., Lauroc.*, and *Puls.* The relation existing between *Cantharis* and *Coffea* is inimical. For profitable comparisons the student is referred to Farrington's *Clinical Materia Medica.*

Edward Fornias, M. D.
711 Pine Street, Philadelphia.

